# Evaluation of Ribavirin–Poloxamer Microparticles for Improved Intranasal Absorption

**DOI:** 10.3390/pharmaceutics13081126

**Published:** 2021-07-23

**Authors:** Dipy M. Vasa, Zainab Bakri, Maureen D. Donovan, Lauren A. O’Donnell, Peter L. D. Wildfong

**Affiliations:** 1 Division of Pharmaceutical, Administrative, and Social Sciences, Graduate School of Pharmaceutical Sciences, School of Pharmacy, Duquesne University, 600 Forbes Ave., Pittsburgh, PA 15282, USA; dipy.vasa@gmail.com (D.M.V.); odonnel6@duq.edu (L.A.O.); 2 Department of Pharmaceutical Science and Experimental Therapeutics, College of Pharmacy, University of Iowa, 115 South Grand Ave., Pharmacy Building, Iowa City, IA 52242, USA; zainab-bakri@uiowa.edu (Z.B.); maureen-donovan@uiowa.edu (M.D.D.)

**Keywords:** ribavirin, poloxamer, microparticles, intranasal delivery, olfactory transport, permeation

## Abstract

Ribavirin is a water-soluble antiviral compound which, owing to its inability to cross the blood–brain barrier, has limited effectiveness in treating viruses affecting the central nervous system. Direct nose-to-brain delivery was investigated for ribavirin in combination with poloxamer 188, an excipient known to enhance the absorption of drug compounds administered intranasally. Composite solid microparticles suitable for intranasal insufflation were prepared by suspending fine crystals of ribavirin in a matrix of poloxamer 188, which were cryogenically milled and characterized to ensure that ribavirin remained stable throughout preparation. In vitro diffusion of ribavirin across a semi-permeable regenerated cellulose membrane showed comparable cumulative drug release after 180 min from both fine solid particles (<20 µm) and 1:1 ribavirin:poloxamer microparticles (d_50_ = 20 µm); however, the initial release from polymer microparticles was slower, owing to gel formation on the membrane surface. When solid ribavirin was directly deposited on excised olfactory mucosa, either as fine drug particles or 1:1 ribavirin:poloxamer microparticles, permeation was significantly increased from microparticles containing poloxamer 188, suggesting additional interactions between the polymer and olfactory mucosa. These data indicate that for highly water-soluble drugs such as ribavirin or drugs subject to efflux by the nasal mucosa, a formulation of poloxmer-containing microparticles can enhance permeability across the olfactory epithelium and may improve direct nose-to-brain transport.

## 1. Introduction

The synthetic nucleoside ribavirin (1-β-D-ribofuranosyl-1,2,4-triazole-3-carboxamide) is a broad-spectrum antiviral drug [1] that has shown promising in vitro activity [2,3,4]. Owing to its high aqueous solubility and low lipid permeability (BCS Class III), ribavirin does not easily cross the blood–brain barrier (BBB), which limits its usefulness against viruses that affect the central nervous system (CNS). This was reinforced by studies showing that intraperitoneal, intramuscular, and subcutaneous ribavirin injections were ineffective against several encephalitis viruses in mice [5,6]. In contrast, intracranial ribavirin administration in virus-infected hamsters allowed for improved survival against subacute sclerosing panencephalitis (SSPE) [6]. This suggests that increasing the CNS bioavailability of ribavirin through alternative routes of administration addresses an important unmet medical need.

Intranasal drug delivery holds promise for CNS targeting, given the potential to bypass the BBB [7,8,9,10,11,12,13]. Direct nose-to-CNS delivery occurs via the olfactory and trigeminal pathways in the nasal cavity, offering several advantages including avoidance of hepatic first-pass metabolism, rapid onset of action, and the potential for patient self-administration.

Ongoing research in this area continues to improve formulations designed to enhance the fraction of dose delivered and retained intranasally [14,15], although devices that enable exclusive drug deposition in the olfactory region are currently unavailable. The performance of liquid formulations is particularly influenced by the size of atomized droplets, where it has been shown that 100% intranasal deposition is possible when diameters exceed 20 µm [16,17,18]. Preferential drug deposition on olfactory tissue has also been shown following breath-actuated insufflation of solid particles smaller than 40 µm in diameter. Prolonged in situ retention of these powders was observed relative to liquid droplets from more traditional sprays [19,20]. It was suggested that the solid particles in contact with the nasal mucus were slowly hydrated, resulting in an increased local viscosity that was more resistant to mucociliary clearance.

Although intranasal residence time is important for successful nose-to-CNS drug delivery, increased CNS uptake also relies on improving permeation of the nasal epithelium. A formulation with select excipients has been demonstrated to be helpful in this regard [21,22]. In a recent study conducted using spray-dried ribavirin as agglomerates with various excipients, enhanced drug permeation across rabbit nasal mucosa was demonstrated for particles that also contained α-cyclodextrin or lecithin, relative to micronized ribavirin particles [23]. Poloxamers, also known as Pluronics^®^, were investigated in the present work. These tri-block-co-polymers consist of polyethylene oxide and polypropylene oxide monomers, and exhibit a thermally reversible sol–gel transition at physiological temperatures. Enhanced permeation across the nasal mucosa has also been shown for formulations containing poloxamers, thought to be a combination of efflux transporter inhibition [24,25], accelerated solute diffusion across the lipid bilayer [26,27], and temporary opening of tight junctions between epithelial cells [28]. As an example, intranasal administration of zidovudine to adult rabbits resulted in CNS concentrations that were nearly four times higher from formulations that contained poloxamer, likely attributable to increased retention in the nasal cavity following polymer gelation, its ability to act as a permeation enhancer, or its inhibition of active drug efflux [29].

Insufflation of solid particles is less common than other means of intranasal administration, including inhalation of atomized liquid sprays or instillation of drops. Consequently, less research has been performed to optimize formulations intended for solid particle insufflation. Although it may be possible to homogeneously mix drug and polymer particles of comparable sizes, the segregation of these components upon administration is undesirable. As such, this study focuses on a formulation in which ultrafine drug particles are suspended in molten polymer, which is solidified by quench cooling. Subsequent micronization retained uniform particle composition while sizing selected particles best suited for intranasal delivery.

The present work details the preparation and characterization of binary composite solid microparticles of ribavirin in poloxamer. An in vitro evaluation of ribavirin permeation across excised olfactory mucosal tissues following delivery from poloxamer-based microparticles was performed to investigate their potential use in intranasal drug administration that directly targets the CNS. Apparent permeation from microparticles was compared to that of both solid drug and aqueous ribavirin solutions.

## 2. Materials and Methods

Solid ribavirin was purchased from Fisher Scientific (Chicago, IL, USA) or from Jinan Jiaquan Chemicals Co. Ltd. (Jinan, China, Lot number 02110330). Poloxamer 188 NF (Pluronic^®^ F68) was purchased from Spectrum Chemicals (New Brunswick, NJ, USA; CAS Number 9003-11-6). The ribavirin and poloxamer used to prepare the microparticles were stored for at least 1 week prior to experimentation in a P_2_O_5_ desiccator (~0% RH) to remove environmental water. Verapamil was obtained from Fisher Scientific (Chicago, IL, USA).

A simulated nasal electrolyte solution (SNES) was prepared for in vitro solubility studies. Consistent with the literature, the SNES combined 8.77 g NaCl, 2.98 g KCl, and 0.59 g anhydrous CaCl_2_ in 1 L deionized water [30]. Solution pH was adjusted with 1 N HCl to simulate the pH of nasal fluid (between 5.5 and 6.0). Permeation studies used Kreb’s Ringer Buffer (KRB), which was prepared by the addition of 0.05 g MgCl_2_, 0.34 g KCl, 7.0 g NaCl, 0.18 g Na_2_HPO_4_, 0.1 g NaH_2_PO_4_, and 1.8 g D-glucose to deionized water. This solution was sparged with carbogen (95% O_2_/5% CO_2_), followed by the addition of 0.28 g CaCl_2_. Bovine mucin was purchased from VWR (Radnor, PA, USA; CAS # 84195-52-8) and added to SNES at 2% *w*/*v* to mimic the mucus secretion in the nasal cavity.

### 2.1. Determination of Ribavirin Solubility in Solid Poloxamer 188

Geometric dilution followed by manual mixing was used to combine small quantities of ribavirin and poloxamer 188. Samples containing 10, 20, 30, 40, 50, 60, 70, 80, and 85% *w*/*w* polymer were analyzed using a Model Q100 differential scanning calorimeter (DSC) (TA Instruments, New Castle, DE, USA). The preparation of 3 replicate samples for each composition was performed by hermetically sealing 5.0 ± 0.5 mg of each mixture in an aluminum pan. Samples were heated at 2 °C/min from ambient temperature to 200 °C. Depressions of the extrapolated onset of melting (*T*_o_), peak maximum temperature (*T*_max_), and enthalpy of melting (Δ*H*_m_) were carefully recorded and used to indicate solubility of ribavirin (*T*_m_ = 168–170 °C) in molten poloxamer 188 (*T*_m_ = 55–60 °C). Results were used to determine maximum drug loading in composite microparticles.

### 2.2. Preparation of Binary Composite Microparticles

A 1 g physical mixture of 1:1 *w*/*w* ribavirin:poloxamer 188 was blended for 20 min at 20 rpm by adding the components to a glass scintillation vial, which was placed in a foam insert inside a jar milling vessel (U.S. Stoneware, East Palestine, OH, USA) that served as a small-scale laboratory blender. Content uniformity was assessed using HPLC (see Section 2.5 below); blends with an RSD <5% with respect to ribavirin were accepted and equilibrated in a P_2_O_5_ desiccator at ~0% RH for 24 h. For 1:1 binary composites, crystalline ribavirin was suspended in molten poloxamer 188 by transferring the components to a crucible, which was immersed in a silicon oil bath maintained at 100 °C, over which dry N_2_ gas was continuously streamed to minimize water vapor sorption. Following mixing, the 1:1 ribavirin:poloxamer suspensions were quenched using liquid nitrogen, which resulted in the formation of a solid disc. Thermogravimetric analysis (TGA) was performed using a Model Q500 instrument (TA Instruments, New Castle, DE, USA). Ribavirin, held isothermally for up to 30 min at 100 °C, underwent <1% weight loss. No DSC events corresponding to degradation or phase transformation at the same temperature were observed, indicating that ribavirin remained thermally stable during the suspension process. Additionally, DSC analyses of the composite confirmed that the ribavirin remained undissolved in the polymer.

Solidified 1:1 ribavirin:poloxamer discs were mechanically size-reduced for intranasal delivery using a cryogenic impact mill (SPEX Certiprep 6750 Freezer Mill, Metuchen, NJ, USA). Approximately 1.0 g of the solid suspension was placed in a cylindrical polycarbonate milling vessel and equilibrated by immersion in the liquid nitrogen bath for 3 min prior to milling. Similar to the preparation method of Crowley and Zografi [31], materials were ground for a total of 20 min at 10 Hz in 2 min intervals, separated by 2 min cool-down periods. Milled samples were sized by sieve fractionation (Performer III Model: SS-3, Gilson Company, Lewis Center, OH, USA), and the particles that passed through the 53 µm sieve (US Standard Mesh No. 270) were collected and stored in a P_2_O_5_ desiccator until further use.

### 2.3. Ribavirin Physical Stability in Poloxamer Microparticles

Cryogenically ground binary microparticles were characterized using powder X-ray diffraction (PXRD) and DSC to ensure that mechanical sizing of the solid suspensions did not result in transformation of crystalline ribavirin to a metastable solid phase [32]. An X’Pert Pro MPD system (PANalytical B.V., Almelo, The Netherlands) was used to generate X-rays at a voltage of 45.0 kV and amperage of 40.0 mA. The instrument was equipped with a Cu anode (λ = 1.5406 Å), an auxiliary elliptical mirror, and an X’Celerator^TM^ detector (PANalytical B.V., Almelo, The Netherlands). Samples were loaded between 2 layers of Kapton^®^ film (ChemPlex, Palm City, FL, USA) and rotated using the vertical transmission stage. The goniometer was set to allow consistent irradiation over 5–50°2θ in 0.017°2θ steps at 135 s/step.

The melting temperatures (*T*_m_) of ribavirin polymorphs were measured using conventional DSC. Hermetically sealed aluminum sample pans containing 5.0 ± 0.5 mg of ribavirin were heated at either 2 °C/min or 40 °C/min to 200 °C [33]. Milled ribavirin:poloxamer microparticles were also analyzed using DSC, and the *T*_m_ for ribavirin in these samples was compared with those of the known polymorphs. All measurements were performed in triplicate and reported as mean values ± SD.

### 2.4. Assessment of Particle Properties

The particle size and size distribution of the 20–53 μm microparticle sieve fraction were determined using a BX-51 optical microscope (Olympus America Inc., Melville, NY, USA) at 10× magnification equipped with a polarizing filter. Crystallinity of ribavirin was confirmed through notable birefringence. Photomicrographs were used to measure the Feret diameter using ImageJ software (version 1.41o, U.S. National Institutes of Health, Bethesda, MD, USA) [34], where measurements were manually selected for approximately 300 particles.

Scanning electron microscopy (SEM) was used to image composite microparticles and their components. A Hitachi S-3400N SEM (Hitachi, Tokyo, Japan) equipped with a Quantax model 400 energy dispersive spectrometer with an XFlash^®^ 5010 EDS detector (Bruker AXS, Berlin, Germany) were used to assess particle morphology. Double-sided carbon tape affixed to an aluminum specimen holder was used to mount uncoated samples. An accelerating voltage of 2 kV, previously demonstrated not to deteriorate uncoated samples during imaging, was used to collect SEM images at a working distance of 15 mm.

### 2.5. High-Performance Liquid Chromatography (HPLC)

A published stability-indicating HPLC method [35] was slightly modified to assay ribavirin. Briefly, a Waters Alliance 2690 LC system equipped with a Waters 996 photodiode array detector and autosampler was used with a 5 μm, 250 mm × 4.6 mm i.d. Hypersil C18 reverse-phase column (Waters Corp., Milford, MA, USA). Isocratic elution of a mobile phase comprised of 0.01 M KH_2_PO_4_ solution and pure methanol (95:5, *v*/*v*) was performed at a flow rate of 1 mL/min. Ribavirin was detected using λ = 207 nm and eluted as a single peak at approximately 4.55–4.65 min.

### 2.6. Microparticle Drug Content and Content Uniformity

Drug content was measured by dissolving a mass of ribavirin:poloxamer microparticles in distilled water, equivalent to a 20 mg dose of drug. The resulting solutions were passed through a 0.45 µm filter and diluted for HPLC analysis. Microparticle content uniformity was determined by random selection of 12 samples from the milled and sized powder. A low variation (RSD < 5%) was chosen as the acceptance criteria for drug uniformity.

Finally, to ensure ribavirin chemical stability during the temperature and mechanically intensive preparation of the solid suspension, the ribavirin was recovered from the poloxamer microparticles by dissolution in a known volume of water. An aliquot from this solution was separated from polymer using a 0.22 µm filter, quantified using HPLC, and compared to concentration from crystalline ribavirin standards. All measurements were reported as mean ± SD.

### 2.7. In Vitro Drug Release

The in vitro ribavirin release studies were performed in triplicate using Franz diffusion cells (PermeGear, Hellertown, PA, USA). A regenerated cellulose dialysis membrane (Spectrum Laboratories Inc., Houston, TX, USA) with a 3000–3500 Da molecular weight cutoff (MWCO) was mounted between the donor and receiver chambers. The receiver compartment was filled with 5 mL of simulated nasal electrolyte solution (SNES), which was maintained at 37 ± 1 °C and agitated using a magnetic stirrer (200 rpm). Either ribavirin solid or ribavirin:poloxamer microparticles were evenly sprinkled on the pre-hydrated dialysis membrane, ensuring that samples contained no more than 5.0 mg of free drug. In order to maintain a hydrated environment similar to that in the nasal cavity, 20 µL SNES containing 2% *w*/*v* mucin was added to the donor side [30]. Then, 200 µL samples were withdrawn at predetermined time intervals using the receiver compartment sidearm, and replaced with an equivalent volume of fresh, pre-warmed SNES. UV spectrophotometry (λ_max_ = 207 nm) was used to quantify the amount of drug released at each time point (Hewlett-Packard 8453 UV-Visibile Spectrophotometer, Agilent Technologies, Santa Clara, CA, USA).

### 2.8. In Vitro Ribavirin Permeation

Ribavirin permeability was measured by adapting the methods published by Chemuturi et al. [36] using bovine olfactory mucosa as a model tissue, sourced from Bud’s Custom Meats (Riverside, CA, USA). The olfactory turbinates were removed within 15 min of decapitation, thoroughly rinsed with Krebs Ringer’s buffer (KRB), and transported in fresh KRB, on ice. To ensure viability, all studies were conducted within 4 h of tissue procurement. The excised olfactory were carefully removed from the underlying cartilage and affixed so that the mucosal side was facing the donor compartment. The pH 6.8 KRB was pre-warmed to 37 °C and added to the receiver chamber (1 mL for vertical cells; 5 mL for horizontal cells). A heating manifold was used to maintain each chamber temperature at 37 °C, while a mixture of 95% O_2_ and 5% CO_2_ was bubbled through the system to ensure oxygenation and agitation.

In the investigation of ribavirin delivery from poloxamer microparticles, the permeability of drug through the bovine olfactory mucosa was measured using a NaviCyte Horizontal Ussing System (Warner Instruments, Holliston, MA, USA) with a cross-sectional permeation area of 0.64 cm^2^. After 30 minutes of equilibration, ribavirin was loaded onto the tissue surface either as a 5 mg/mL aqueous solution, 5 mg of ribavirin solid, or 10 mg of 1:1 ribavirin:poloxamer microparticles. For the solid sample test groups, an additional 50 µL of KRB was added to the tissue surface to mimic the volume of secretions in the nasal cavity.

At predetermined time points over the following 2 h interval, samples (250 µL for horizontal cells, 200 µL for vertical cells) were withdrawn from the receiver compartment and replaced with an equal volume of fresh, pre-warmed KRB. The amount of permeated drug was determined using UV-visible spectrophotometry at λ_max_ = 207 nm (for horizontal cells) or by HPLC (for vertical cells) using an Agilent 1100 apparatus (Agilent Technologies Co., Santa Clara, CA, USA) with a Waters Symmetry C18 (3.9 mm × 150 mm; 5 µm) column (Waters Corporation, Milford, MA, USA) and a 95:5 0.1M KH_2_PO_4_:methanol mobile phase. Ribavirin absorbance was detected at 207 nm.

Equation (1) was used to calculate the apparent permeability coefficient, *P*_app_ (cm/s):

(1) $$P_{\mathrm{app}}=\frac{\left(\frac{dM}{dt}.\;\frac{1}{A}\right)}{C_{0}}\times \;60$$

where d*M*/d*t* (µg/min) is the rate of mass transport obtained from linear regression of the steady-state portion of a plot of the cumulative amount of drug in the receiver cell, *M* (µg), at time, *t* (min). The cross-sectional area, *A*, was constant for permeability experiments (0.64 cm^2^), and *C*_0_ was recorded as the initial donor concentration (µg/mL).

### 2.9. In Vitro Nasal Cell Toxicity

Eagle’s minimal essential medium (MEM), supplemented with 10% fetal bovine serum (FBS), 2 mM L-glutamine, 100 units/mL penicillin, and 100 mg/mL streptomycin, was used to grow RPMI 2650 human nasal septal carcinoma cells (ATCC cat. no. CCL 30), between passages 27 and 32, in collagen-coated T-75 flasks (Novagen Inc., Darmstadt, Germany). The medium was humidified in a 37 °C incubator with 5% CO_2_ and changed every 36–48 h. Upon reaching 80–90% confluency, a 0.1% trypsin-EDTA solution was added, and the cells were re-plated in collagen-coated 96-well plates (Novagen Inc., Darmstadt, Germany) at a density of 3 × 10^4^ cells per well in 200 µL culture medium and incubated overnight for cell attachment. Subsequent removal of 100 µL of culture medium from the wells and exchange for solutions of ribavirin, poloxamer 188, or a 1:1 *w*/*w* ribavirin and poloxamer 188 mixture in MEM resulted in final concentrations of 0, 7.8, 15.6, 31.25, 62.5, 125, 250, 500, and 1000 µg/mL of each component, with further incubation for 24 h.

Cell viability was determined by reducing the medium volume in each well to 50 µL and adding 25 µL of CellTiter-Glo^®^ reagent (Promega, Madison, WI, USA). Mixing for 15 min on an orbital shaker was used to induce cell lysis, and following 10 min incubation at room temperature, the medium was transferred to a polystyrene assay plate (Costar #3912, Corning, NY, USA), where luminescence was measured using a Model 1420-051, Victor3 microplate reader (Perkin Elmer, Shelton, CT, USA).

## 3. Results and Discussion

### 3.1. Preparation and Characterization of Binary Composite Microparticles

Composite microparticles were designed to contain phase-pure inclusions of crystalline ribavirin suspended in a solid poloxamer 188 matrix to enable intimate mixing between the two components and alleviate segregation issues likely to occur with a simple physical mixture. Rapid solidification of the polymer resulted in a suspension of phase-pure API crystallites surrounded by a re-solidified polymer matrix, as demonstrated previously [37]. Cryogenic milling of ribavirin for longer than 60 min has been shown to result in polymorphic changes of the drug [32], so the total milling time was limited to shorter durations (i.e., 10 or 20 min).

Each DSC thermogram showed two distinct melting endotherms consistent with the values for poloxamer 188 (*T*_m_ = 57.07 ± 1.31 °C) and the desired polymorph of ribavirin (*T*_m_ = 166.87 ± 2.03 °C) [33]. Of note, neither the melting onset (*T*_o_) nor the peak maximum temperature (*T*_max_) for the microparticles deviated significantly relative to either pure component, indicating that little to no drug dissolved in the molten polymer during preparation and confirming that a suspension of ribavirin existed in the solidified poloxamer 188 matrix. Avoiding partial or complete dissolution of ribavirin in the molten polymer was desired in order to prevent heterogeneous formation of some molecularly dispersed drug surrounded by variable degrees of suspended crystalline ribavirin, which would likely result in variable delivery, dissolution, and physical stability. These data also suggested that a relatively high fraction of ribavirin could be suspended in poloxamer 188, allowing the formulation to accomplish the need for high drug loading while still providing sufficient poloxamer to promote permeation enhancement. Based on preliminary studies, a formulation containing 50% *w*/*w* drug-loaded poloxamer, which was milled for 10 min, provided a good yield of the microparticles in the desired size range, with content uniformity <5% RSD, and this formulation was used for subsequent experiments.

**Figure 1:**
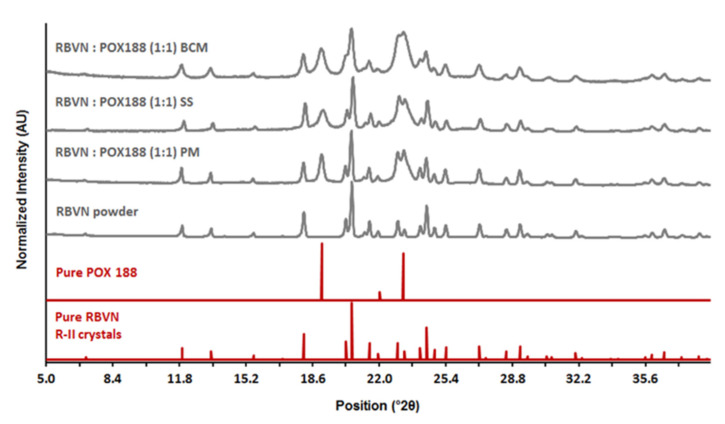
PXRD patterns of 1:1 ribavirin (RBVN) and poloxamer (POX188) during different stages of suspension preparation: PM—physical mixture, SS—solid suspension, BCM—binary composite microparticles after sieve fractionation. PXRD patterns of pure components are provided for comparison.

Following preparation and milling, PXRD and DSC were used to characterize materials. Figure 1 compares the PXRD patterns of ribavirin powder, poloxamer 188, and processed microparticles. Characteristic Bragg peaks for ribavirin at 11.94, 18.15, 20.3, 20.6, 24.44, and 27.1°2θ, and for poloxamer at 19.1, 22.0, and 23.2°2θ, all remained consistent relative to the reference diffractograms for both physical mixtures and milled microparticles. These data indicate that microparticle preparation did not change the crystal forms of either component.

DSC results for the 1:1 composite microparticles showed two distinct endothermic peaks at 55.9 ± 1.59 °C and 168.2 ± 0.48 °C, which were attributable to poloxamer 188 and ribavirin, respectively. DSC thermograms for the microspheres were similar to those for the physical mixtures, showing no reduction in the melting of poloxamer 188 and a 3 °C depression in the onset of ribavirin melting, a slight difference that was likely caused by particle size reduction. None of the microparticles showed any evidence of a solid form change, confirming that ribavirin and poloxamer 188 remained phase-pure. When a rapid-heating rate DSC (40 °C/min) was also used to improve detection of small quantities of either amorphous ribavirin or the metastable polymorph R-I, neither was observed.

### 3.2. Particle Properties

**Figure 2:**
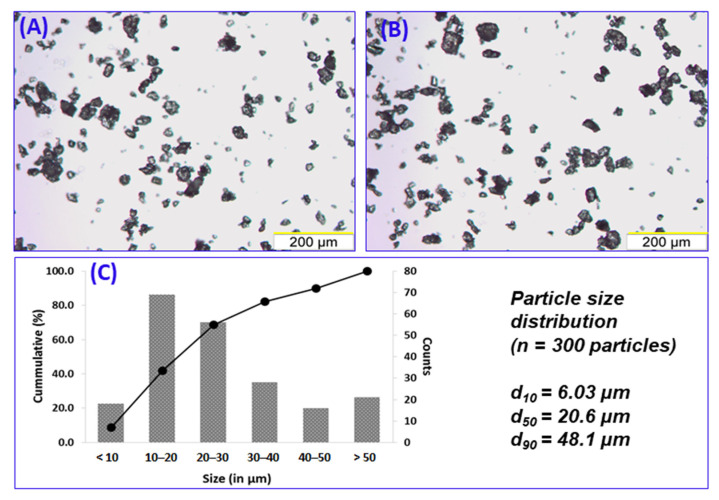
Representative micrographs (10 × objective) depicting particle size distribution of 1:1 ribavirin:poloxamer microparticles (**A**,**B**). Measurement of Feret diameter (*n* = 300) of the particles resulted in the particle size distribution shown in (**C**).

The selected sieve fraction (<53 μm) of ribavirin:poloxamer microparticles was characterized for particle size distribution and bulk morphology (Figure 2A,B). The median primary particle size (*d*_50_) was 20.6 µm, with *d*_10_ and *d*_90_ respectively measuring 6.03 µm and 48.1 µm (Figure 2C). When observed under polarized light, particles were prominently birefringent, indicating that the suspended drug was crystalline (data not shown).

**Figure 3:**
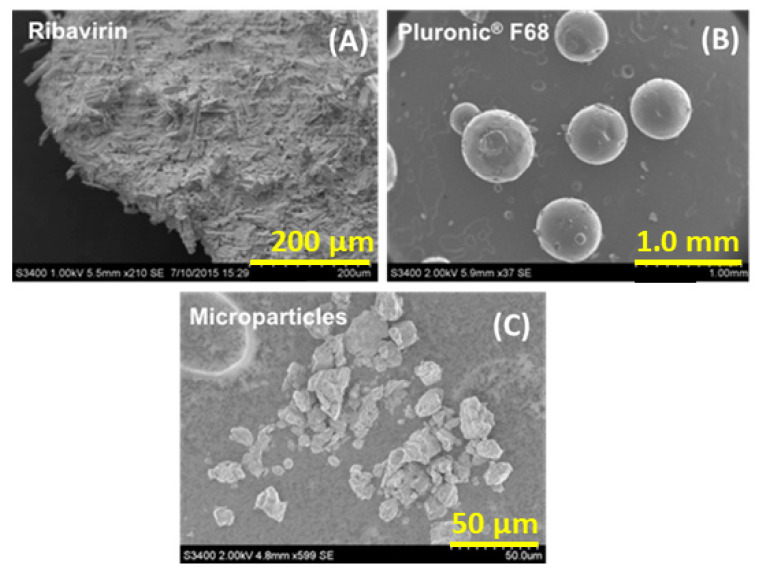
Scanning electron micrographs: (**A**) unprocessed ribavirin, (**B**) Pluronic^®^ F68 (poloxamer 188), and (**C**) 1:1 (*w*/*w*) ribavirin:poloxamer microparticles (sized by sieving < 53 μm).

The bulk morphology of the final microparticles was assessed using SEM imaging and compared with the components from which they were made. Figure 3 shows that the microparticles were irregularly shaped relative to the larger agglomerates of ribavirin needles and the much larger poloxamer 188 spherical particles. While the rough texture of the microparticles shown here is not typically suited to administration from nasal devices, it is expected that at larger scales, the necessary size reduction step will likely result in a more regular particle morphology, making the resulting composite particles more appropriate for insufflation and nasal deposition.

### 3.3. Binary Composite Microparticle Performance

#### 3.3.1. In Vitro Drug Release

The in vitro release of ribavirin from composite microparticles was evaluated using a Franz diffusion cell equipped with a semi-permeable regenerated cellulose membrane. Selective permeation of the dissolved ribavirin molecules (244.21 Da) was facilitated by a 3000–3500 Da MWCO, which retained the poloxamer 188 (~8400 Da) on the donor chamber membrane surface. In contrast to typical diffusion experiments, the volume available for dissolution was purposefully maintained at 20 µL on the donor side to mimic the hydration volume typical of the nasal mucosa in an adult human. Consequently, in vitro ribavirin release was considered to consist of four steps: (1) formation of a drug-containing poloxamer gel resulting from microparticle hydration, (2) dissolution of the drug within the hydrated polymer, (3) diffusion of the ribavirin, first through the polymer matrix, and then through the cellulose membrane into the receiver, and (4) accumulation of ribavirin in the receiver compartment.

**Figure 4:**
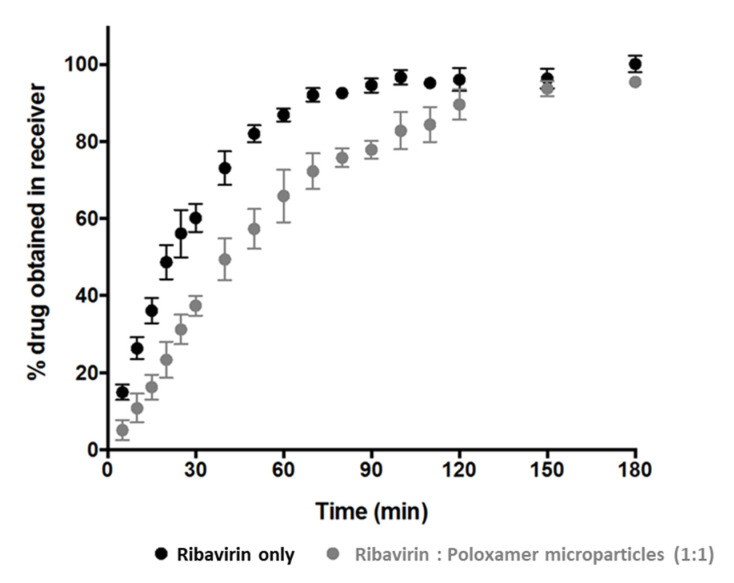
The apparent cumulative ribavirin release from solid drug particles (black circles) and ribavirin:poloxamer microparticles (grey circles).

Owing to the complexity associated with this release method, additional investigations using fine ribavirin particles (sieve fraction 20–53 µm) were conducted to identify the influence of the barrier presented by the poloxamer matrix. The results indicate that the dissolution of ribavirin is not instantaneous, and yet approximately 70% of the drug dissolved and permeated through the cellulose membrane in 45 min and ~97% was in the receiver chamber after 180 min (Figure 4).

Although the extent of cumulative drug release at 180 min from ribavirin:poloxamer microparticles remains comparable to the ribavirin particle dissolution, the initial release rate from the gel formed by the microparticles was somewhat slower than that from the ribavirin particles. This was not unexpected, since the drug in the microparticles must first dissolve and then diffuse through the polymer–gel matrix prior to diffusing through the cellulose membrane, which would be expected to decrease the rate at which ribavirin appears in the receiver compartment. As the release experiments progressed, rapid conversion of solid ribavirin:poloxamer microparticles into a thin film over the cellulose membrane surface was observed. The slower drug release from the ribavirin:poloxamer microparticles (Figure 4) was likely due to the increased viscosity and matrix properties of the poloxamer gel observed to be forming on the surface of the cellulose acetate membrane. Several reports suggest that poloxamer-containing gel formulations can be retained in the nasal cavity for up to 100–110 min [29,38], which suggests adequate time for drug release from ribavirin:poloxamer microparticles may be possible in the nasal cavity.

#### 3.3.2. In Vitro Cytotoxicity

**Figure 5:**
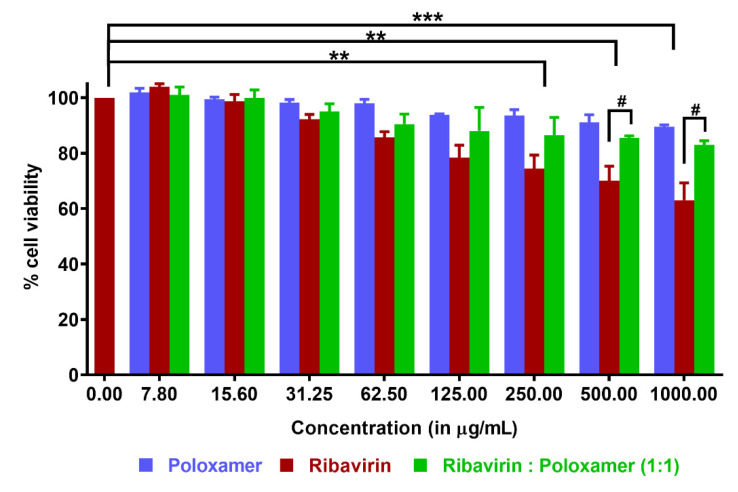
Cytotoxicity assay using CellTiter Glo^®^ reagent (reported as mean ±SD, *n* ≥ 3): RPMI 2650 cells were incubated with varying concentrations of poloxamer, ribavirin, and a 1:1 ribavirin:poloxamer mixture. Statistical differences within the same group are denoted as ** *p* < 0.01 and *** *p* < 0.001, and those between groups are denoted as # *p* < 0.05.

Pre-clinical studies that assess transmucosal drug transport or excipient toxicity can be performed during the development of drug-loaded formulations for intranasal administration using excised tissue, primary cell cultures, or immortalized cell lines [39]. RPMI 2650, is a commercially available immortalized cell line derived from an anaplastic squamous cell carcinoma of the human nasal septum, which resembles normal human nasal epithelium both morphologically and biochemically [39,40,41,42]. Cell viability was quantified using RPMI cells growing in 96-well plates following a 24 h incubation with ribavirin, poloxamer 188, and a 1:1 mixture of drug and polymer over a spectrum of concentrations (Figure 5). As expected, increasing ribavirin concentrations on RPMI 2650 cells resulted in a greater reduction in cell viability, consistent with its known inhibition of the synthesis of intracellular DNA, resulting in cell death and prevention of division [43,44]. In the test group containing only the drug at concentrations of 250, 500, and 1000 μg/mL, the measured cell viability was respectively reduced by 26%, 30%, and 37%, respectively.

No cytotoxicity was observed for poloxamer 188 solutions over the range of concentrations studied, which agreed well with previous observations in other cell lines [45,46]. In fact, the cytoprotective effect of poloxamers has been demonstrated both in vitro and in vivo [47,48]. The ability of poloxamer 188 to repair damaged cell membranes is well-documented; although this occurs by mechanisms that are not entirely clear, repair may be attributable to direct polymer incorporation into the phospholipid bilayer, which helps increase the lipid packing density [49]. In the present study, cell viability was improved considerably when the cells were exposed to ribavirin in the presence of poloxamer 188, as compared to cells exposed to ribavirin alone at the same concentration. This improvement was even more pronounced at higher concentrations of the drug. For example, at 1000 μg/mL, cells incubated with only ribavirin retained 63% viability, while this was improved to 90% in the presence of poloxamer 188.

#### 3.3.3. Ribavirin Permeation through Olfactory Mucosa

**Figure 6:**
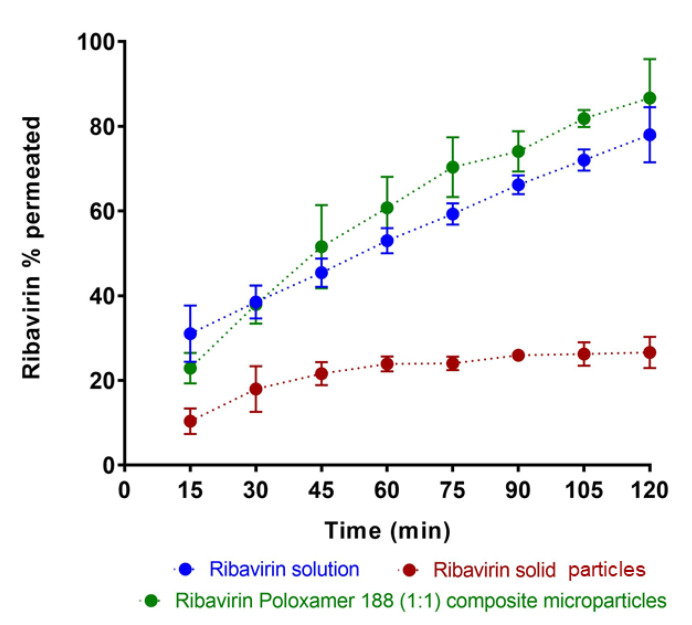
A comparison of in vitro permeation profiles of ribavirin across bovine olfactory tissue when loaded as ribavirin solution, 1:1 ribavirin:poloxamer microparticles, and pure solid ribavirin particles. Data are expressed as mean ± SD, *n* = 3.

The present work was designed to evaluate ribavirin permeation from solid composite microparticles when deposited directly on nasal mucosal tissues. A comparison of the permeation profiles for ribavirin from different test groups is shown in Figure 6. When a 0.2 mL aliquot of a 5 mg/mL aqueous solution of ribavirin was added to the donor compartment of the NaviCyte Horizontal Ussing System (tissue exposed to a total of 1 mg of drug), the apparent permeability coefficient (*P*_app_) was estimated to be (1.34 ± 0.17) × 10^−5^ cm/s, with a linear increase in ribavirin in the receiver cell indicative of steady-state diffusion across the tissue. Notably, these data agreed closely with a previously reported value of *P*_app_ for ribavirin across rabbit nasal mucosa (1.92 ± 0.18) × 10^−5^ cm/s [50].

For a comparison with aqueous ribavirin solution, the direct deposition of 1.0–1.5 mg solid drug particles was attempted. Owing to difficulties in placing only 1 mg of particles on the tissue surface, a slightly greater amount (5 mg) of ribavirin solid was directly deposited on the mucosal surface, followed by the addition of 50 µL KRB, resulting an effective initial concentration of 100 mg/mL. Under these conditions, not all of the drug immediately dissolved owing to the unstirred environment on the tissue surface. Instead, the smallest drug particles likely dissolved quickly, and were responsible for the initial rapid mass transport of ribavirin over the first hour of the permeation study.

To provide a comparable drug exposure in the donor compartment, 10 mg of 1:1 ribavirin:poloxamer microparticles were deposited on the tissue (i.e., 10 mg of 1:1 binary microparticles contains 5 mg of drug). Notably, when the drug was introduced to tissue as 1:1 ribavirin:poloxamer microparticles, ribavirin dissolution was likely enhanced, and drug permeation was greater than for an aqueous solution of the drug, despite the increased viscosity of the poloxamer gel medium.

Ribavirin permeation was expected to be similar to that observed in the in vitro release studies, owing to the similarity in the experimental methods, except for the change in membrane from dialysis cellulose to bovine olfactory mucosa. The significantly increased permeation of ribavirin from the poloxamer microparticles suggests additional interactions between the formulation and the olfactory mucosa that contribute to the improved absorption of drug.

In addition to its thermal gelation properties, poloxamer 188 has also been reported to inhibit p-glycoprotein and other efflux transporters, many of which have been reported to be present in the olfactory mucosa [24,25,26,51,52]. Poloxamer 188 has also been shown to have direct effects on membrane fluidization [49] and can induce transient opening of tight junctions [53], which both act to increase membrane permeability. While ribavirin is a known substrate for both concentrative and equilibrative nucleoside transporters (respectively, CNT and ENT), including CNT2 and CNT3 along with ENT1 and ENT2 [54,55,56], it has not been reported to be a substrate for any of the ABC-efflux transporters. Similar nucleoside drugs have been reported to act both as p-glycoprotein substrates and inhibitors [57,58], so further evaluation of the interaction of ribavirin with p-glycoprotein was undertaken. 

Using NaviCyte Vertical Ussing Chambers, ribavirin permeation across the nasal olfactory mucosa was investigated in the presence of verapamil, a classic p-glycoprotein inhibitor. Bakri previously demonstrated that ribavirin showed concentration-dependent permeation across olfactory tissues [59], and when a 1.2 mg/mL ribavirin solution was tested, the drug permeability was found to be 1.3 × 10^−5^ cm/s. However, when verapamil (50 µM) was included, the permeability of ribavirin increased nearly 2-fold to 2.6 × 10^−5^ cm/s. These results clearly demonstrate that ribavirin interacts with a variety of transporters, including p-glycoprotein, and the ability of formulation excipients to modulate those transporters can result in significant increases in ribavirin transport across the olfactory epithelium.

**Figure 7:**
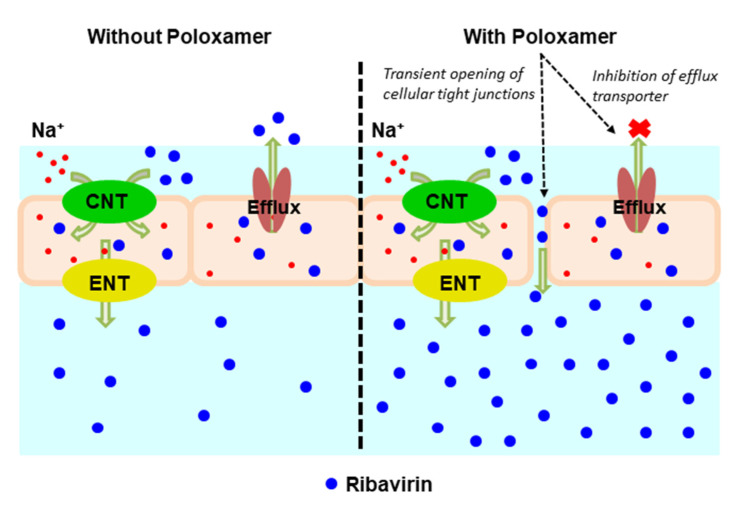
Schematic representation of ribavirin (blue closed circles) transport across the olfactory epithelium. (Left panel) In the absence of poloxamer (POX188), permeation is enabled by concentrative nucleoside transporters (CNT) and equilibrative nucleoside transporters (ENT), ultimately limited by drug efflux by p-glycoprotein. (Right panel) In the presence of POX188, the transient opening of tight junctions and inhibition of p-glycoprotein efflux increase the overall permeation of ribavirin.

Figure 7 shows a schematic representation of the proposed mechanisms for ribavirin transport across the olfactory epithelium. Due to its hydrophilicity, the passive transport of ribavirin is expected to be low and much of its permeation is likely the result of the action of transporters located within the epithelium. The 1:1 ribavirin:poloxamer microparticles improve the absorption and delivery of ribavirin across the nasal mucosa by several complementary mechanisms, including increased permeability due to the combination of transient opening of tight junctions and local poloxamer action on the epithelial cells, decreased transporter-mediated efflux of ribavirin, and the reduced cytotoxicity associated with high ribavirin concentrations.

## 4. Conclusions

Solid microparticles comprised of a 1:1 suspension of fine crystalline ribavirin in a poloxamer 188 matrix were developed and tested for their ability to improve nasal absorption drug. Binary ribavirin:poloxamer microparticles milled to a median size of 20 μm were found to be physically and chemically stable, with a narrow particle size distribution and precise content uniformity. The extent of ribavirin release from binary microparticles was comparable to that from fine particles of the drug, but permeation of ribavirin across excised bovine olfactory tissues was the greatest from the polymer microparticles containing poloxamer 188. These results suggest that the formulation of a poorly permeable drug, such as ribavirin, with an active excipient, such as poloxamer 188, can provide significant improvements in the overall delivery of the drug, both due to the effects of the polymer on drug permeability and the added potential for an increased nasal residence time resulting from the formation of a viscous gel on the mucosal surface as the particles dissolve.

## Data Availability

All of the data described in this report is included in the publication or available in the cited references.
